# Diagnosis and Management of Struma Ovarii in Pregnancy: A Case Report

**DOI:** 10.3390/life15081328

**Published:** 2025-08-21

**Authors:** Isidoro Narbona Arias, Lucia Castaño Frías, María Marfil Gonzalez, Laura Baños Cárdenas, Jesús S. Jimenez Lopez

**Affiliations:** 1 Obstetrics and Gynecology Department, Hospital Materno-Infantil, Hospital Regional Universitario Málaga, Avenida Arroyo de los Ángeles S/N, 29011 Málaga, Spain; dr.narbona@gmail.com (I.N.A.); laurabc84@hotmail.es (L.B.C.); jesuss.jimenez@uma.es (J.S.J.L.); 2 Research Group in Maternal-Foetal Medicine Epigenetics Women’s Diseases and Reproductive Health, Biomedical Research Institute of Malaga (IBIMA), 29010 Málaga, Spain; 3 Department of Surgical Specialties, University of Malaga, 29010 Málaga, Spain

**Keywords:** struma ovarii, adnexal tumor, ovarian torsion, pregnancy

## Abstract

Adnexal tumors during pregnancy are rare, with a prevalence ranging from 0.05% to 3%, and in most cases, they are benign. Struma ovarii, a monodermal teratoma, consists of over 50% thyroid tissue and accounts for 2.7% of ovarian teratomas. It typically affects women aged 40–60 and is exceptionally rare during pregnancy. Diagnosis is often only established after surgical intervention and histological examination. We present the case of a 39-year-old pregnant woman (gravida 2, para 1) at 19 weeks of gestation who presented with acute lower abdominal pain. At her first visit at 11 weeks, ultrasound revealed a 12 cm multilocular left adnexal mass. Initial conservative management was followed by emergency laparoscopy due to suspected ovarian torsion, resulting in a left oophorectomy. Histopathology confirmed struma ovarii. Thyroid function tests (TSH, FT4) remained within normal limits throughout pregnancy. The pregnancy continued without further complications, culminating in a spontaneous vaginal delivery at 40 + 4 weeks of a healthy female infant weighing 3800 g. Due to the rarity of this condition, treatment guidelines remain undefined, with management decisions relying on limited case reports and clinical judgment. This report highlights the importance of detailed evaluation and individualized management in such uncommon presentations during pregnancy.

## 1. Introduction

According to the World Health Organization, ovarian neoplasms are classified by tissue of origin into three main groups: 70% derive from coelomic epithelium, 20% from germ cells, and 10% from sex cords [1]. Teratomas, which originate from germ cells, are characterized by tissues derived from the three embryonic germ layers. Struma ovarii is a monodermal variant of ovarian teratoma in which more than 50% of the tumor is composed of thyroid tissue. The first documented case was reported by Boettlin in 1889 [2].

Struma ovarii accounts for 2.7% of all ovarian teratomas [3,4]. It is usually unilateral, most often affecting the left ovary, with bilateral involvement in approximately 6% of cases. It can occasionally be associated with other ovarian tumors such as cystadenomas or mature teratomas in the contralateral ovary [4]. The condition most commonly affects women between 40 and 60 years of age [2]. Malignancy is rare, occurring in 5–10% of cases, with papillary and follicular carcinomas being the most common malignant subtypes [3,4,5,6].

Adnexal masses during pregnancy are uncommon, reported in 0.2–3% of cases, a figure that has increased with routine prenatal ultrasound [7,8,9]. These lesions are usually benign, with dermoid cysts being the most frequent. Struma ovarii is particularly rare in pregnancy [8]. Clinical presentation varies widely: 20–44% of patients are asymptomatic, while others may experience pelvic pain, ovarian torsion, or ascites (notably in malignant cases) [10]. In 5–8% of cases, patients may present with hyperthyroidism due to autonomous hormone production [9,10].

Preoperative diagnosis of struma ovarii is challenging, often established only after histopathological examination following surgery [8]. The objective of this manuscript is to present a detailed case report of struma ovarii complicated by ovarian torsion during pregnancy, emphasizing diagnostic difficulties, management decisions, and outcomes to provide practical guidance for clinicians facing similar rare scenarios.

## 2. Case Description

**Figure 1:**
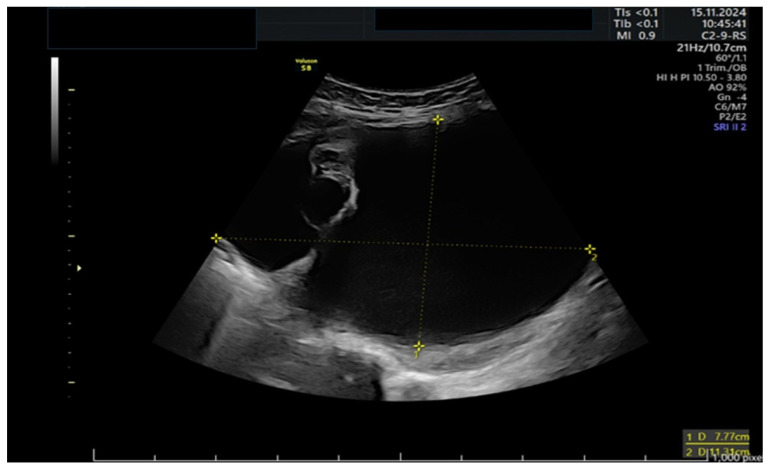
Ultrasound of a large 12 cm tricameral adnexal mass without Doppler signal.

**Figure 2:**
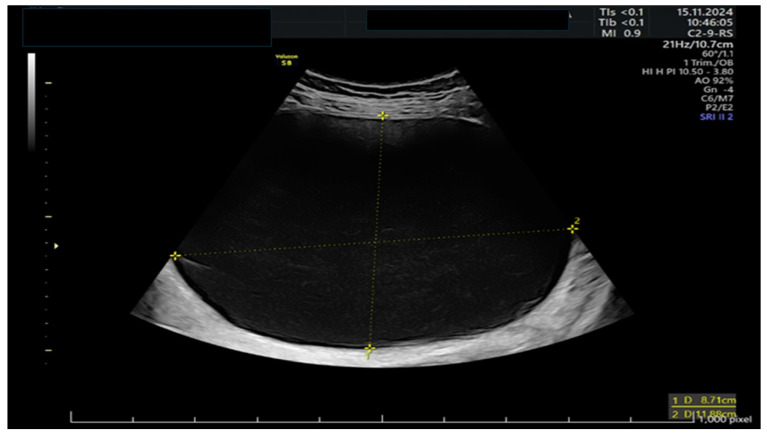
Detail of the major cystic compartment on ultrasound.

A 39-year-old woman presented to the emergency room at 11 weeks of gestation with lower abdominal pain. She had no relevant medical or surgical history, as well as a previous low-risk gestation, and her current pregnancy had been uneventful. At that time, she was known to have a left ovarian cyst regularly followed-up with serial ultrasounds. The bedside ultrasound scan revealed a 12 cm multilocular left adnexal mass, with thin walls and anechoic content, no papillae, and a color score of 1, as shown in Figure 1 and Figure 2. There were no signs of ovarian torsion at that time and after clinical improvement, the patient was discharged from the hospital with an arranged follow-up appointment. Tumor markers were requested, and normal results were yielded for CA 125 (16), CA 15.3 (11), and CA 19.9 (8).

**Figure 3:**
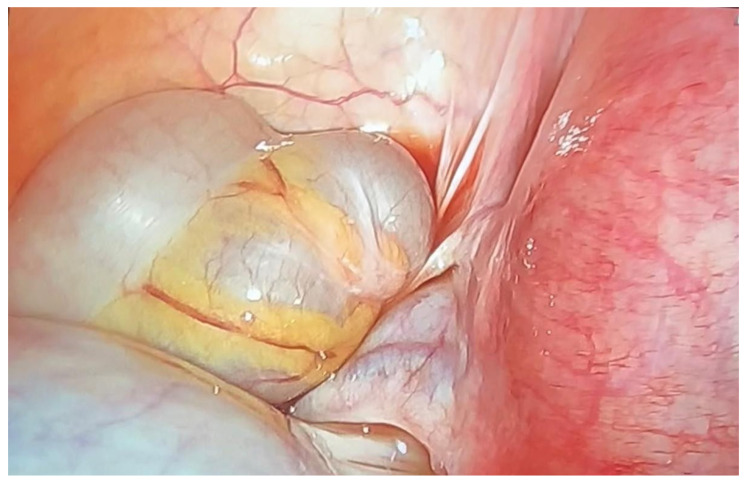
Detail of adnexal tumor formation with torsion.

Nevertheless, the patient reattended the emergency room at week 19, presenting with acute onset of left lower quadrant pain. In this case, the ultrasound examination showed a positive whirlpool sign and on suspicion of ovarian torsion, the patient underwent an emergency diagnostic laparoscopy. Intraoperative findings revealed a 12 cm mass and twisting of two turns at the left infundibulopelvic ligament (Figure 3).

Consequently, a left oophorectomy was performed. The patient was discharged from hospital the following day, with no signs of immediate postoperative complications. Subsequent routine medical appointments were found to be normal, with no indications of complications, and the fetus was growing in accordance with its gestational age. A histological examination of the tissue removed during the operation confirmed a diagnosis of struma ovarii.

Prior to surgery, thyroid function tests revealed normal levels of TSH (1.620 µUI/mL) and free T4 (14.38 pmol/L), indicating no biochemical thyroid dysfunction at baseline. Following the intervention and throughout the remainder of the pregnancy, thyroid hormone levels (TSH, T3, and T4) remained within normal parameters upon serial analytical control, further supporting the absence of functional impact from the ovarian struma.

At 40 weeks and 4 days of pregnancy, the patient was admitted to hospital due to prelabor rupture of the membranes. The patient underwent a straightforward vaginal delivery, resulting in the birth of a 3800 g female infant, and both mother and baby were discharged from hospital following a 48 h stay.

The patient has been under the care of the Gynaecology and Endocrinology teams since giving birth, with all visits to date having been satisfactory.

## 3. Discussion

The incidence of adnexal lesions during pregnancy has increased concomitantly with the development of ultrasound technology. The majority of these lesions are benign and asymptomatic and are typically diagnosed during the first trimester, with 75% of cases resolving spontaneously [7,10].

As the IOTA (International Ovarian Tumor Analysis) ultrasound evaluation algorithms have not been validated for use during pregnancy, an approximate study based on the Simple Rules model is advised to distinguish between benign and malignant lesions. In the present case, the adnexal lesion demonstrated features suggestive of malignancy (multilocular structure with a diameter exceeding 10 cm). In cases where ultrasound findings remain inconclusive, the use of magnetic resonance imaging (MRI) may be useful as a secondary imaging tool [7] and to evaluate the extent of the disease in cases of suspected or confirmed malignancy [11].

The reliability of tumor markers is reduced in pregnant patients when compared to non-pregnant patients. Levels of the marker CA-125 can be elevated at the commencement of the first trimester of pregnancy and subsequently return to normal levels during the second and third trimesters. However, in certain cases, the levels can remain persistently elevated in healthy patients [11,12,13,14,15,16]. Consequently, its use is not recommended during pregnancy, as this reduces its reliability in distinguishing between malignant and benign lesions [7].

Struma ovarii is a monodermal teratoma characterized by the presence of thyroid tissue comprising over 50% of the tumor, and it accounts for 2.7% of ovarian teratomas. It is most commonly unilateral, with the left ovary being typically affected, as evidenced in the case study presented.

It has been observed that this condition is most prevalent during the first two trimesters of pregnancy, as seen in the current case. A significant proportion of patients remain asymptomatic, with approximately 8% developing symptoms of hyperthyroidism. In this patient, thyroid function had not been evaluated prior to diagnosis, as struma ovarii was not initially suspected. However, the TSH value measured at the routine first-trimester antenatal check-up was within the normal range.

In some cases, the diagnosis is only established when complications occur, which happen in 2% to 5% of cases. Ovarian torsion is a relatively common complication, particularly in cases involving lesions measuring between 6 and 10 cm, which is consistent with the presented case, and it is characterized by an acute onset of abdominal pain [17].

Following the diagnostic process, the most suitable management option for the patient must be determined. In asymptomatic pregnant women with benign-appearing lesions, a conservative ‘wait-and-see’ approach with periodic follow-up examinations may be considered. In the case presented, this approach was initially adopted, with scheduled periodic follow-up appointments, as the patient was asymptomatic [17]. However, it is important to note that this management strategy carries certain risks, such as rupture or torsion.

In cases where surgical intervention is deemed necessary, it is recommended to perform the intervention between 16 and 18 weeks of gestation, unless an acute complication such as rupture or torsion occurs, in which case surgery should be performed immediately [7,17].

Despite the ongoing controversy regarding the most suitable surgical approach for pregnant patients, laparoscopy was preferred over laparotomy in this case due to its well-documented advantages, including reduced postoperative pain, a lower need for analgesia, and shorter hospital stay, as highlighted by the work of Ye et al. [16,17].

**Figure 4:**
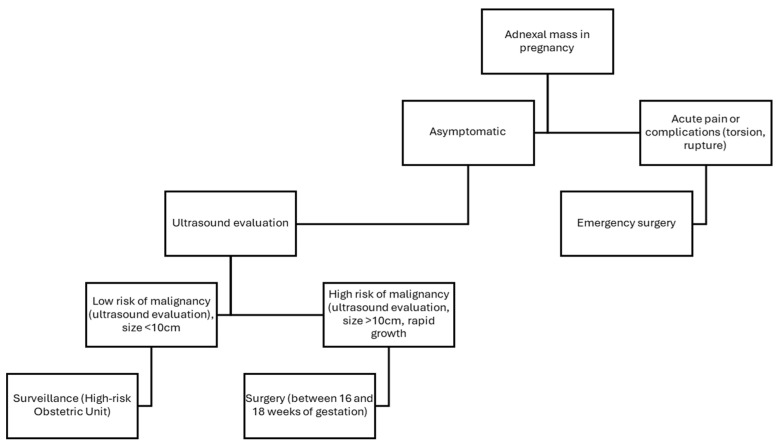
Proposed management of adnexal masses during pregnancy.

Preoperative planning during pregnancy is of paramount importance. Precise knowledge of uterine size and lesion dimensions is crucial to appropriately adjust patient positioning and trocar placement, ensuring a safe and effective surgical approach in each case [7] (Figure 4).

## 4. Conclusions

The incidence of adnexal lesions has increased alongside the widespread use of prenatal ultrasound, which has enabled an earlier detection of these masses. Struma ovarii accounts for only a small percentage of ovarian teratomas and is exceptionally rare in pregnant patients. The condition typically presents as an asymptomatic adnexal mass, although complications such as ovarian torsion, as seen in this case, may arise, particularly in larger masses.

The acute onset of abdominal pain in the patient at 19 weeks of gestation prompted surgical intervention, which is consistent with the recommended management approach for adnexal torsion. Minimally invasive laparoscopic surgery was successfully performed, reinforcing the growing body of evidence supporting its safety and efficacy during pregnancy. The advantages of laparoscopy, including reduced operative blood loss, shorter hospital stays, and faster recovery, make it an appealing option for managing adnexal masses when surgery is required.

Despite the progress made in understanding the management of adnexal masses during pregnancy, there is still no clear consensus on the optimal treatment strategies for rare conditions such as struma ovarii. The limited number of documented cases and the lack of well-defined prognostic markers hinder the development of standardized guidelines. As a result, treatment decisions must be tailored to the individual patient’s clinical presentation, necessitating close monitoring and a multidisciplinary approach. Ongoing research and continued case reporting are crucial to improving our understanding of this condition and developing effective treatment strategies.

## Data Availability

The original contributions presented in the study are included in the article; further inquiries can be directed to the corresponding author.
